# In silico analysis of binding interaction of conantokins with NMDA receptors for potential therapeutic use in Alzheimer’s disease

**DOI:** 10.1186/s40409-017-0132-9

**Published:** 2017-09-20

**Authors:** Maleeha Waqar, Sidra Batool

**Affiliations:** Department of Biosciences, COMSATS Institute of Information Technology, Park Road, Chak Shahzad, Islamabad 45550 Pakistan

**Keywords:** N-methyl-D-aspartate, Glutamate, Synaptic plasticity, NR2B, Neurotransmitter, Antagonists, Conantokins, Docking, In silico, Alzheimer’s dieases

## Abstract

**Background:**

The N-methyl-D-aspartate (NMDA) receptors are glutamate receptors that play vital roles in central nervous system development and are involved in synaptic plasticity, which is an essential process for learning and memory. The subunit N-methyl D-aspartate receptor subtype 2B (NR2B) is the chief excitatory neurotransmitter receptor in the mammalian brain. Disturbances in the neurotransmission mediated by the NMDA receptor are caused by its overexposure to glutamate neurotransmitter and can be treated by its binding to an antagonist. Among several antagonists, conantokins from cone snails are reported to bind to NMDA receptors.

**Methods:**

This study was designed to analyze the binding mode of conantokins with NMDA receptors in both humans and rats. To study interactions, dockings were performed using AutoDock 4.2 and their results were further analyzed using various computational tools.

**Results:**

Detailed analyses revealed that these ligands can bind to active site residues of both receptors as reported in previous studies.

**Conclusions:**

In light of the present results, we suggest that these conantokins can act as antagonists of those receptors and play an important role in understanding the importance of inhibition of NMDA receptors for treatment of Alzheimer’s disease.

## Background

The N-methyl-D-aspartate (NMDA) receptors are inotropic glutamate receptors that are gated cation channels [1, 2]. The NMDA receptors (NMDAR) play vital roles in central nervous system (CNS) development [2]. These receptors are highly permeable to Ca^2+^ ions and the calcium flux is critically important for synaptic plasticity, which is an essential neurochemical process for learning and memory [2–4]. The receptor itself has many subunits and their variants have numerous functions in the brain. The subunit N-methyl-D-aspartate receptor subtype 2B (NR2B) is the chief excitatory neurotransmitter receptor in the mammalian brain [5]. The glutamate neurotransmitter allows for a transmembrane ion flow through the receptor to increase the action potential of the neuron. This characteristic makes the synapsis among these neurons to be the main memory storage unit and hence associates them with learning and memory [6]. Due to their functioning in the CNS, the potential of these receptors as drug targets for various neurodegenerative diseases has been highlighted in the literature.

NMDAR antagonists have emerged as potential lead compounds for Alzheimer’s patients [7]. The cognitive symptoms associated with deficits in learning and menory have been attributed to disturbances in glutaminergic neurotransmission [8]. The excessive stimulation by the glutamate neurotransmitter of neurons causes excitotoxicity and results in damage and death of neurons [8]. Blocking the glutaminergic neurotransmission mediated by NMDA receptors can alleviate the excitotoxicity and prevent further neuron damage and death.

Several venom toxins have made their way in scientific studies and clinical trials for their therapeutic potential against various diseases. Having mostly inhibitory effects, these toxin peptides target different receptors across the body, the hyperactivity of which is associated with the pathophysiology of many diseases. Numerous toxins have been reported to target and block receptors used for the treatment of Alzheimer’s disease, such as neurotoxins that target acetylcholinesterase enzymes and certain toxins from Mamba snakes that were reported to inhibit the muscarinic acetylcholine receptors [9, 10]. A few toxins with antagonistic properties against NMDA receptor have been reported as well. The conantokins are powerful and potent blockers of the NMDA receptor, with particularly high selectivity for the NR2B subunit [11]. These toxins have been reported to block these glutamine receptors and therefore have therapeutic potential for treating Alzheimer’s disease.

The objective of this study was to perform and look into the in silico analysis of the binding interaction of conantokins with the NMDA receptor NR2B subunit. The mode of interaction and the binding residues for both the ligand dataset and the receptor dataset were collected. Due to unavailability of the crystal structures of the NMDA receptor in humans and most of conantokins, their three-dimensional structures were predicted via computational homology modeling methods and the predicted models were validated to continue their further use.

Docking studies provided insights into binding pattern of receptors and ligands. A number of in silico studies investigated many computational approaches ranging from construction of structural models to investigation and discovery of potential drug candidates [12–14]. The analysis of the binding interactions of the receptor and the ligand peptides produced results that helped us demonstrate the pharmacological importance of conantokins and their potential use as NMDA receptor antagonists for treatment of Alzheimer’s disease.

## Methods

### Receptor dataset collection

The first step of methodology included collection of receptor proteins. As the structure of NMDA receptor in humans is not available, it had to be predicted via computational homology modeling, which allowed the construction of a three-dimensional structure of a protein based on the known structures of similar protein templates. SWISS-MODEL was used for this purpose, as it is a fully automated protein structure homology modeling server [15]. The protein template used for structure prediction of NMDAR in humans was the structure of NMDAR in *Rattus norvegicus* (brown rat) [16]. The structure of the template (pdb id: 3JPW) was retrieved from the Research Collaboratory for Structural Bioinformatics (RSCB) [17]. After structure prediction, the Structure Analysis and Verification Server (SAVES) was used for validation by generating Ramachandran plot [18] and ERRAT [19], which gives a factor of overall quality of the predicted structure. Whereas Verify_3D [20] was employed to analyze the compatibility of the atomic model of the protein with its own amino acid sequence.

### Ligand dataset collection

**Fig. 1:**
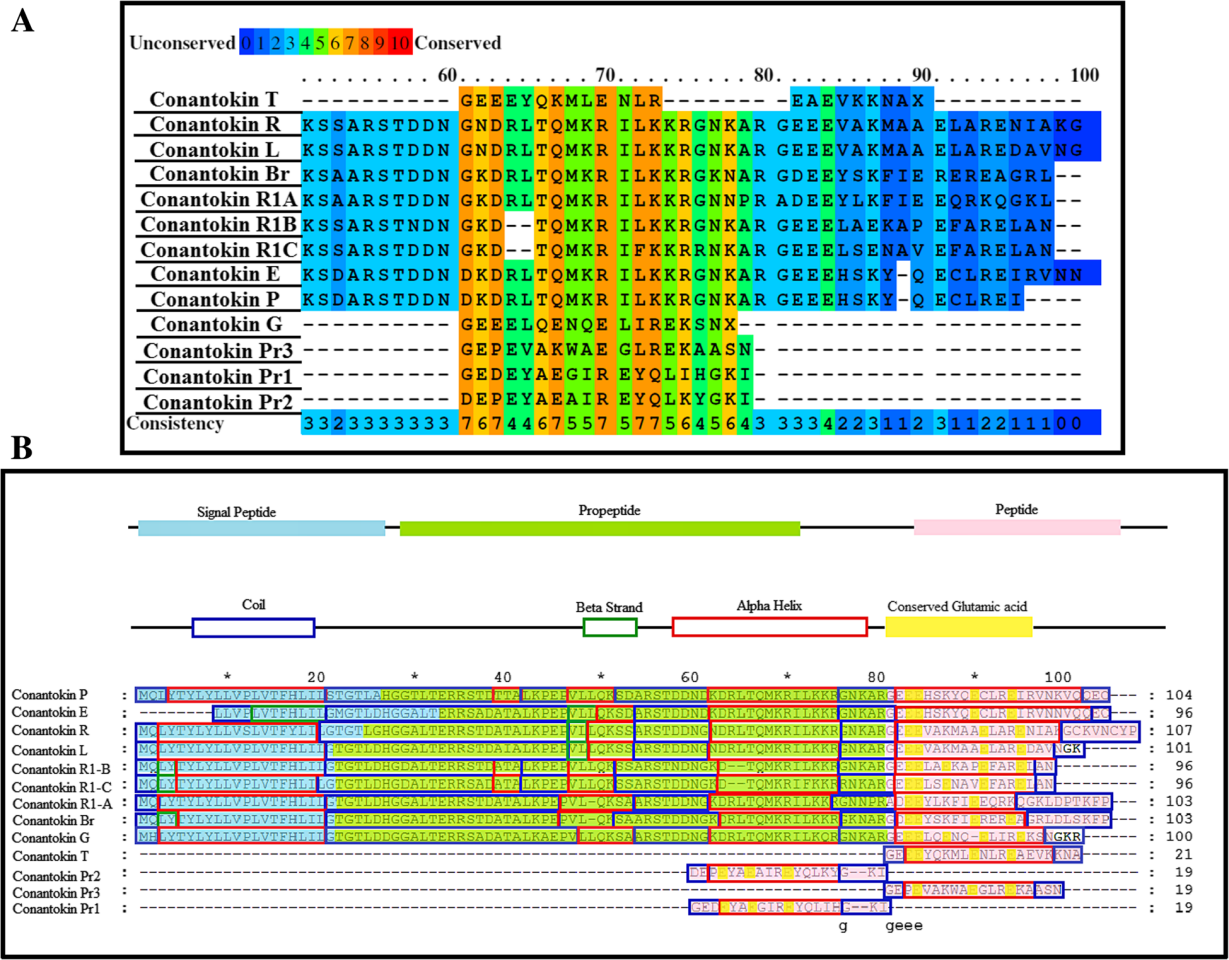
**a** Conserved residues highlighted in conantokins. **b** Predicted secondary structures of all conantokins aligned

The ligand dataset comprised reported conantokins that are a class of conopeptides (17–27 amino acids) without cysteine residues that selectively influence NMDA receptors [21]. Among them, only three-dimensional structures of conantokin G and conantokin T are available on Protein Data Bank (PDB ID: 1ONU and 1ONT, respectively). The crystal structures of other conantokins Br, L, P, R, E, Pr1, Pr2, Pr3, R1A, R1B and R1C are not available on the Protein Data Bank and therefore were subjected to three-dimensional structure prediction. Homology modeling was used initially, but due to absence of any homologues for the conantokins, the structure prediction was carried out via fold recognition (threading) on the Iterative Threading ASSEmbly Refinement (iTASSER) server [22], which detects structure templates from the Protein Data Bank and constructs full-length structure models by reassembling structural fragments from threading templates. The predicted structures were then validated on the SAVES metaserver using Procheck, ERRAT and Verify_3D. Multiple sequence alignment (MSA) using PRALINE [23] multiple sequence alignment toolbox was performed on all the conantokins to find out residues and secondary structure conservation. The residues responsible for the signal peptides, peptide precursors and active protein peptides were also highlighted for each conantokin showing considerable conservation in these peptide regions as well, as shown in Fig. 1.

The conserved glutamic acid residues in each conantokin are the positions for residue modification, where the glutamic acid is converted to 4-carboxyglutamate. Sufficient conservation of secondary structures was observed among all the conantokins.

### Experimental background reported for conantokins and NMDAR

Conantokins have been the subject of interest for their potential as NMDA receptor antagonists in studying many neuropathologies. Wet lab experiments have been conducted to understand the basis for interaction between conantokins and NMDA receptor. These wet lab techniques were carried out using NMDA receptors in rats by employing electrophysiological techniques to understand the diversity in the functioning of these toxins [24]. Techniques such as polyamine enhancement and NMR spectroscopy have allowed the understanding of how similar the inhibition of NMDAR by conantokins in humans is to previously reported inhibition of NMDAR in rats by conantokins [25]. Using chemically synthesized conantokin variants (created by techniques of point mutation and chimeric proteins), the subtype selectivity of NMDAR has been extensively studied for most conantokins [26]. In addition, conantokins represent a class of NMDA antagonists with an improved safety profile compared to other antagonists that show psychotomimetic, amnesic and motor-impairing actions and neurotoxicity, which limit their usefulness in humans [21]. These wet lab studies revealed the pharmacological importance of conantokins as antagonists of NMDAR for their therapeutic applications in many neuropathologies and have led us to investigate this property computationally.

### Docking studies

Molecular dockings are performed to predict the binding orientation between a receptor and its ligand to form a stable molecular complex [27]. This allows a detailed insight of the three-dimensional structure formed between two biomolecules and to correlate this information to find a potential drug candidate for these receptors [28]. Molecular docking was performed for NMDA receptors for both humans and rats. AutoDock 4.2 [29] was used to perform automated docking runs, in order to find the binding mode of each receptor with each conantokin ligand. The number of runs for each docking was set to 50 to allow each ligand 50 different conformations with the receptors in order to let them bind freely anywhere on the receptor. The grid size was set to cover the entire receptor in order to find a potential binding site for each ligand and to analyze if each ligand indeed occupies the site on the receptor that is crucial for its functioning in Alzheimer’s and could potentially inhibit it. The docking parameters used are shown in Table 1.

**Table 1:** Docking parameters used for docking studies on AutoDock 4.2

Grid Parameters		Docking Parameters	
Spacing	0.375 Å	Energy evaluations	2.5 × 10^6^
Grid center	80X Å	Iterations	27,000
	80Y Å	Mutation rate	0.02
	80Z Å	Crossover rate	0.80
		Elitism value	1
		RMS Tolerance	1.0 Å

Post-docking analysis of the results was carried out on AutoDock 4.2 and later visualized on Chimera [30]. Ligplot^+^ [31] was used for further validation of the protein-protein interactions, which generates two-dimensional schematics on the basis of hydrogen bonds and hydrophobic interactions. The three-dimensional structural analysis was performed on PyMol [32] that allowed the residues in the protein-protein interaction of the receptor-ligand complex to be highlighted. The energy of each complex that was achieved via docking allowed the receptor-ligand bindings to be confirmed and refined and used for further analysis and results discussion.

## Results and discussion

### Structure prediction and validation of NMDAR in human

**Fig. 2:**
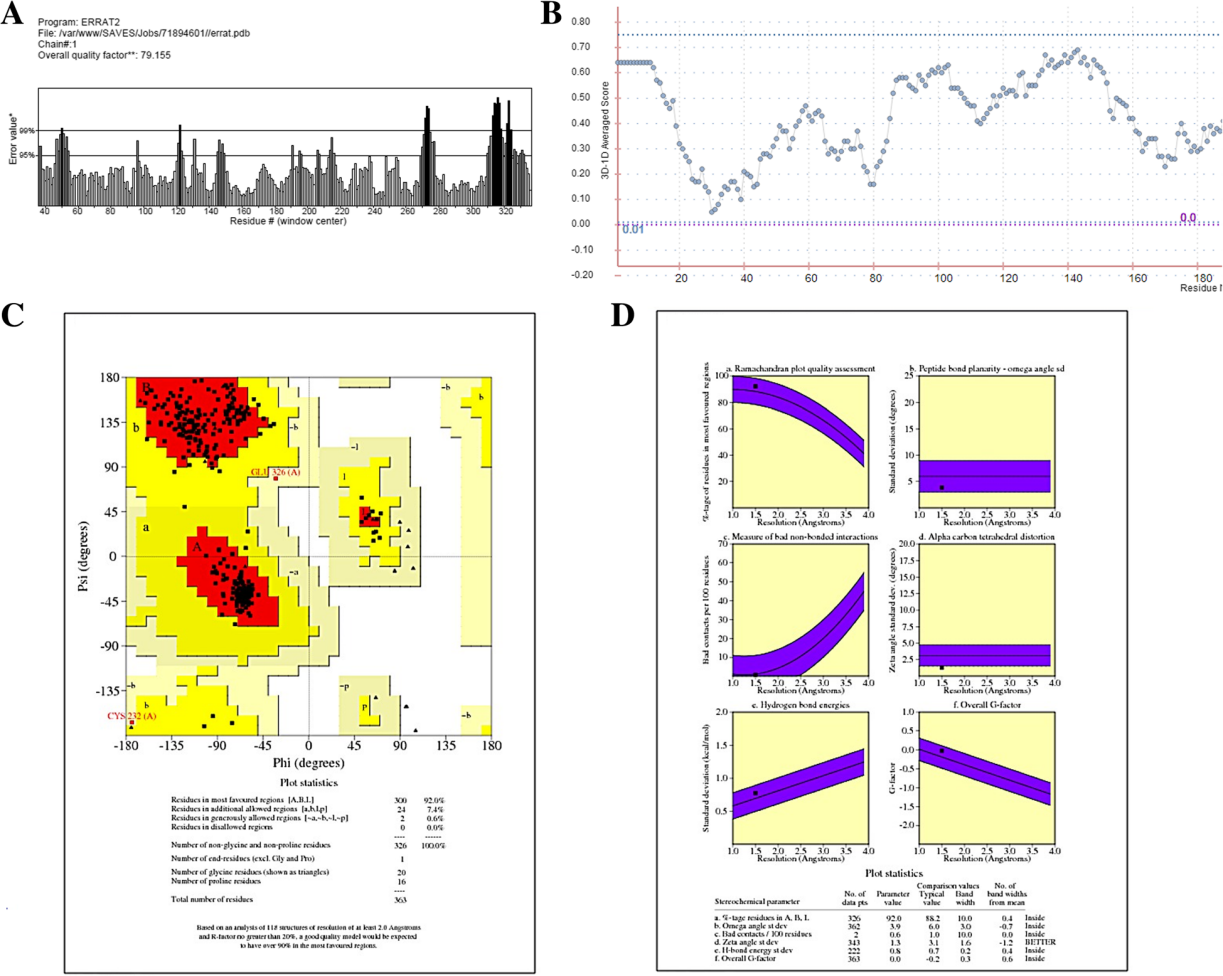
Validation results for the predicted structure of NMDAR in humans. **a** Graphical representation of results from ERRAT. **b** Graphical representation of results from Verify_3D. **c** Ramachandran Plot for NMDAR (humans). **d** Main chain parameters

The predicted three-dimensional structure of the NMDA receptor in humans was superimposed on Chimera with its template, a NMDA receptor in rat yielding a root mean square deviation (RSMD) of 0.181 Å. In addition, the results for structure evaluation proved the quality of the predicted structure. As indicated by the Ramachandran plot, 92% of the residues were in favored regions. Moreover, the main chain parameters such as peptide bond planarity, measure of non-bonded interactions, α carbon tetrahedral distortion, H-bond energy and overall G factor for the structure were found within favorable regions. ERRAT and Verify_3D further validated the structure by scoring it with an overall quality factor of 79.155 and by passing it with 80% of the amino acids having scored ≥0.2 respectively (Fig. 2). The validation results suggested that the predicted model of the NMDA receptor in humans was of good quality and can be used for further analysis.

### Structure prediction and validation of conantokins

The predicted structure of each conantokin was also validated in order to check the quality of their three-dimensional structures. All predicted structures were passed by Ramachandran plot, ERRAT and Verify_3D, suggesting that these structures were of good quality and could be used further study.

### Binding site residue information

**Fig. 3:**
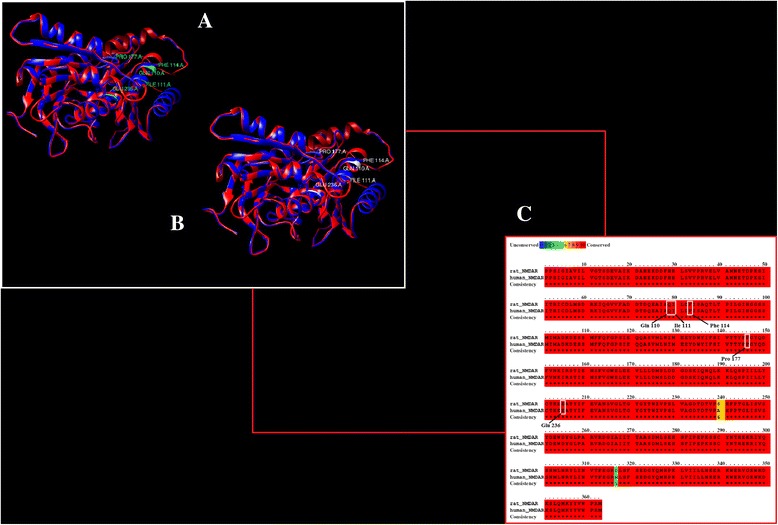
**a** NMDAR in humans (red) active residues highlighted in green. **b** NMDAR in rats (blue) residues aligned with residues of NMDAR in humans highlighted in white. **c** Conserved residues shown in sequence alignment between the NMDA receptor in rats (above) and humans (below)

After structure prediction and evaluation, binding site residue information for NMDA receptor in humans was gathered. Since this is a computational based analysis, it is very important to identify binding site residues correctly and verify the results. As no previous information for binding residues of NMDAR in humans was reported, the binding site was retrieved by using the binding site residues of NMDAR reported in the literature that showed residues specific for the NR2B subunit in rats. The two structures were aligned and it was observed that the binding site residues reported for NMDAR in rats are conserved in humans. The binding residues for NMDAR in both humans and rats are shown in Fig. 3. These identified residues have been taken as references to further evaluate the docking results.

Binding residue information for conantokin G only suggests Glu2, Gln6, Asn8, Arg13, Asn17 and Lys15 as predominant residues that are involved in interactions with the NMDAR subtype selectivity for NR2B subunit. Moreover, the first five amino acids of conantokins, especially Glu2 and Gla4 and a hydrophobic residue at position 12 are critical for functional activity in vitro [21]. It has been observed that the peptide region has shown importance in interactions. These residues are also found to be conserved in other conantokins as well.

### Docking results

After structure prediction and binding site identification, molecular dockings were performed on NMDAR receptors with conantokin ligands. Docking results were run based on their energy values and were ranked as such with each run showing results for receptor-ligand complex with the lowest energy. Detailed analysis of each run for each complex showed that all the conantokins showed bindings with the conserved active sites of NMDA receptor in both humans and rats. All the conantokins seemed to bind with the glutamine and glutamic acid residues in the NMDA receptors in both humans and rats. Table 2 shows the detailed analysis retrieved from plotting the docking results on LigPlot^+^, showing the binding of NMDA receptor in humans with conantokins. These data include information about respective hydrogen bond residues of both receptors and ligands, their bond distances, bond atoms and the interacting hydrophobic residues.

**Table 2:** Docking results of conantokin ligands in complex with NMDA receptor in humans

Conantokin	Hydrogen bonds				Hydrophobic bonds	
	Receptor residues	Ligand residues	Distance (Å)	Atoms	Receptor residues	Ligand residues
Conantokin G	–	–	–	–	Glu236 Gln110 Phe114 Ile111 Pro177	Ile12 Leu5 Ser16 Gln9
Conantokin L	Gln110	Asp96 Ala93 Leu65	2.36 2.25 2.81	N-OD1 OE1-N NE2-O	Pro177 Ile111 Glu236 Phe114	Met88 Thr24 Val85 Gly81
Conantokin E	Pro177 Glu236	Arg67 Asp18 Gly13 Gly12	3.09 2.32 2.23 3.19	O-NH1 OE2-N N-O OE2-N	Ile111 Phe114 Gln110	Leu41 Gln59 Lys66
Conantokin Pr1	Glu236	Gln13	3.13	OE2-NE2	Pro177 Gln110	Asp3 Ala6
Conantokin Pr2	Glu236	Lys15	3.11	OE2-NZ	Gn110 Ile111 Phe114	Glu4 Ala8 Tyr5
Conantokin Pr3	–	–	–	–	Gln110 Ile111 Phe114 Pro177	Glu2 Gly1 Glu4 Trp8
Conantokin T	Glu236 Gln110	Arg13 Gln6	2.85, 2.92 2.88	OE2-NH2 OE2-NE OE1-NE2	Ile111	Glu16
Conantokin R1A	Glu236 Gln110	Arg54 Asp58	2.70 2.82	O-NH1 N-O	Ile111 Phe114 Pr0177	Leu13 Leu18 Glu33
Conantokin R1B	Gln110	Tyr8	3.22	N-OH	Ile111 Pro177 Glu236	Lys62 Lys71 Ala95
Conantokin R1C	Gln110	Val11	2.06	NE2-O	Glu236 Ile111 Pro177 Phe114	Asp77 Ala39 Pro12 Leu13
Conantokin R	Glu236 Phe114	Gln50 Ala98	3.33 2.01	OE2-NE2 N-O	Ile111 Pro177 Gln110	Ser52 Leu65 Met68
Conantokin Br	Gln110	Ala94	1.93	NE2-O	Pro177 Ile111 Glu236	Thr22 Ile82 Asp77
Conantokin P	Glu236	Leu65	2.33	N-OE2	Gln110 Ile111 Phe114 Pro177	Arg80 Glu45 Lys43 Val47

**Fig. 4:**
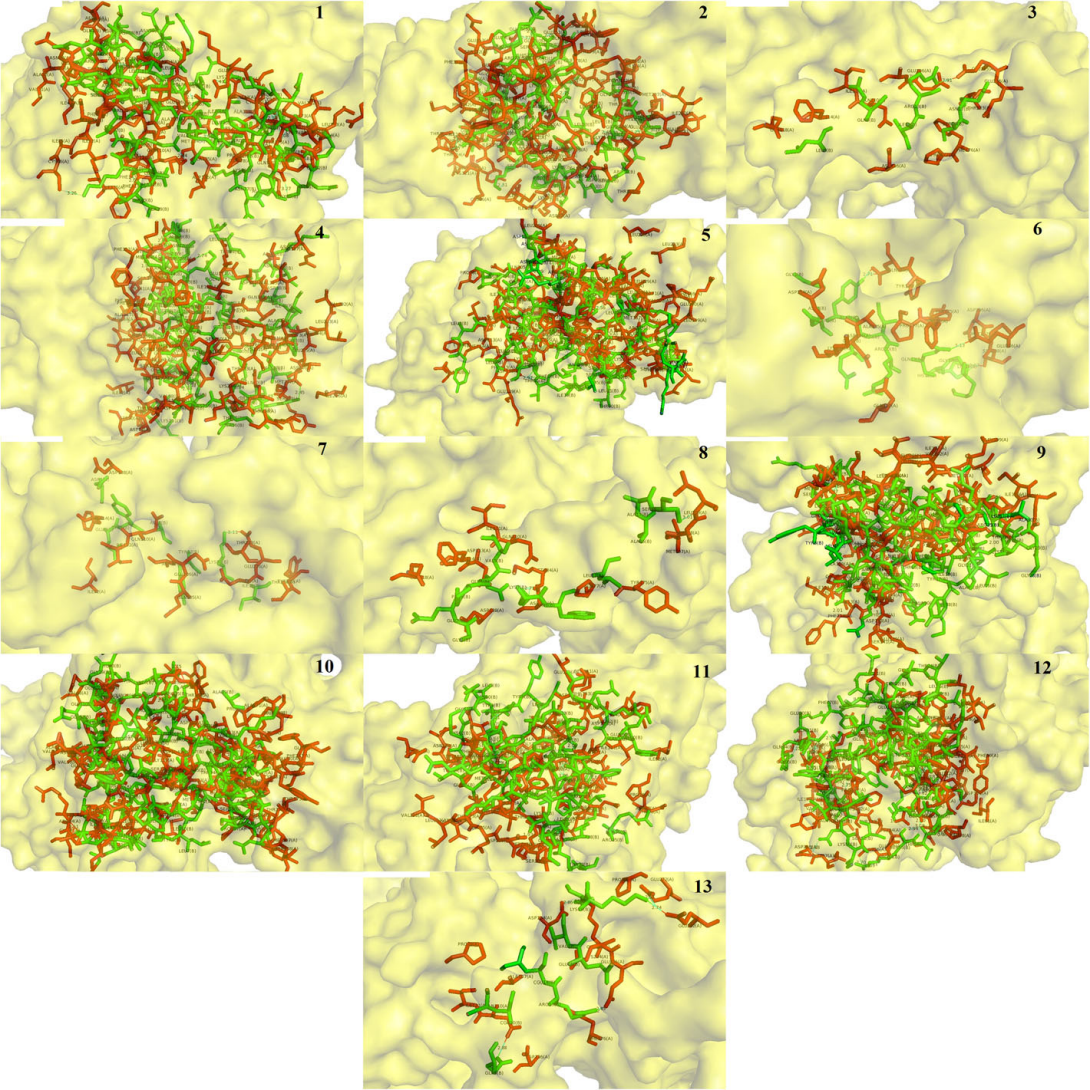
Three-dimensional binding representation of NMDAR (humans) with each conantokin ligand: (**1**) conantokin Br, (**2**) conantokin E, (**3**) conantokin G, (**4**) conantokin L, (**5**) conantokin P, (**6**) conantokin Pr1, (**7**) conantokin Pr2, (**8**) conantokin Pr3, (**9**) conantokin R, (**10**) conantokin R1B, (**11**) conantokin R1C, (**12**) conantokin R1A, (**13**) conantokin T. Receptor chain (red), ligand chain (green), ligand-receptor complex surface (yellow)

Glutamic acid at position 236 and glutamine at position 110 were predominantly involved in the binding of the NR2B subunit of NMDAR in human with all the conantokins. Isoleucine at position 111, phenylalanine at position 114 and proline at position 177 were mostly found in hydrophobic interactions. The binding patterns of all the conantokin ligands individually in complex with the NR2B subunit of NMDAR in human are shown in Fig. 4.

**Fig. 5:**
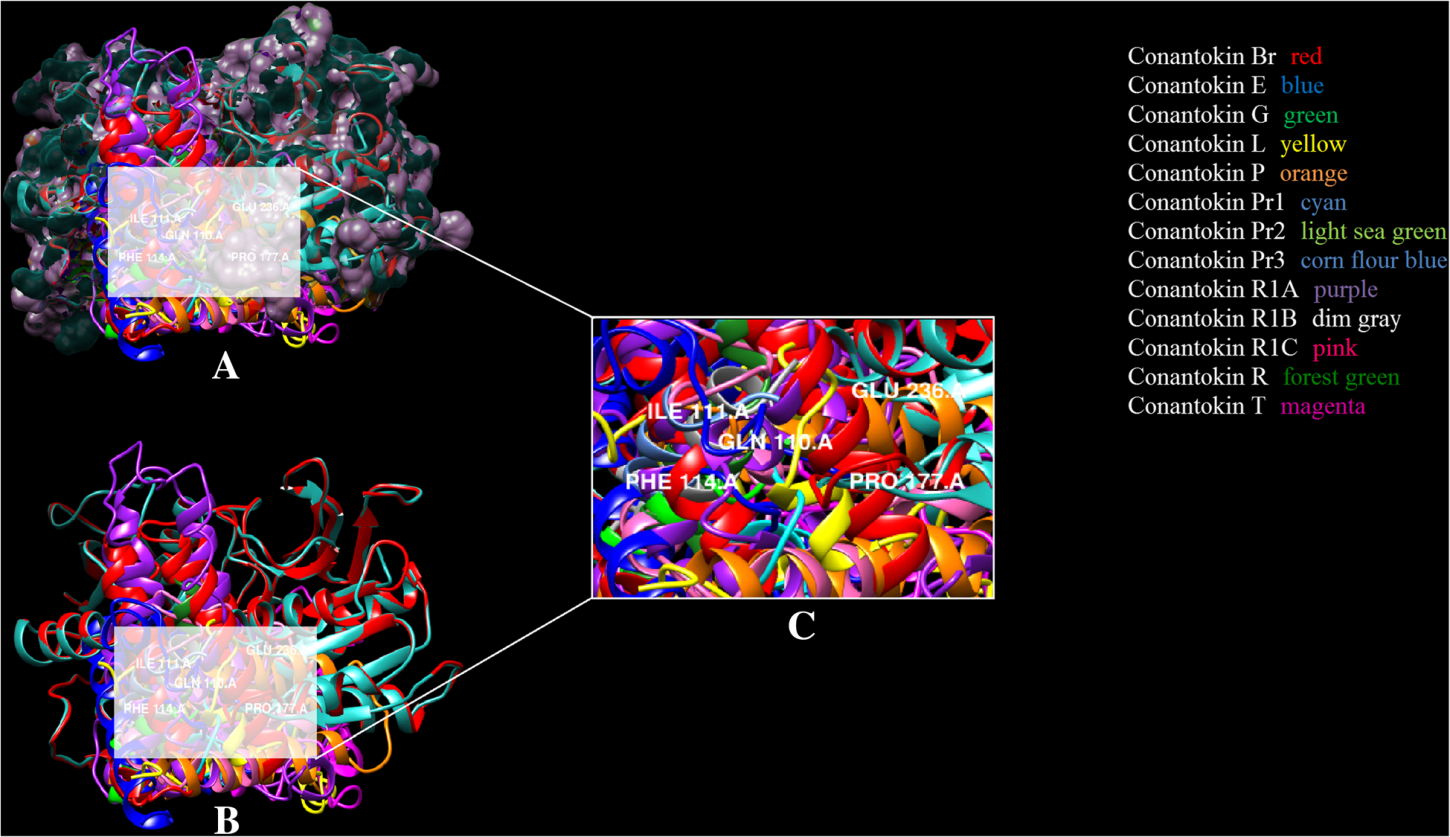
**a** Conantokins in complex with the NMDAR (humans). **b** Active residues in the binding pocket highlighted (white) of the NMDAR with conantokin ligands. **c** Binding pocket highlighted and focused. Three-dimensional binding representation of all toxins from the ligand dataset: conantokin Br (red), conantokin E (blue), conantokin G (green), conantokin L (yellow), conantokin P (orange), conantokin Pr1 (cyan), conantokin Pr2 (light sea green), conantokin Pr3 (cornflour blue), conantokin R1A (purple), conantokin R1B (dim gray), conantokin R1C (pink), conantokin R (forest green), conantokin T (magenta) with the NMDA receptor in human

It is clear in Fig. 4 that due to difference in sizes and structure, each conantokin occupies the binding site on NMDAR in humans in its own respective orientation to form the most stable complex. However, each ligand is shown to bind to the same binding residues as reported. The position of binding pocket of the NR2B subunit of NMDAR in humans resided by conantokins is shown in Fig. 5. It is shown clearly that all the ligands occupied the same binding pocket in structure of NMDAR as they showed binding with the same residues that have been reported.

Similar binding pattern was observed for the NR2B subunit of NMDAR in rat. Table 3 shows the binding information for NMDAR in rats, highlighting residues involved in hydrogen bonding, their atoms and their bond distances, along with the hydrophobic residues for both the receptor and the ligands.

**Table 3:** Docking results of conantokin ligands in complex with NMDA receptor in rats

Conantokin	Hydrogen bonds				Hydrophobic bonds	
	Receptor residues	Ligand residues	Distance (Å)	Atoms	Receptor residues	Ligand residues
Conantokin G	Gln110	Arg13	2.64	OE1-NE	Ile111 Phe114	Ile12 Gln9
Conantokin L	Gln110	Pro46	1.52	NE2-O	Ile111 Phe114 Pro177 Glu236	Pro12 Ala30 Asp77 Val42
Conantokin E	Gln110 Ile111	Ile11 Cys83	2.94 1.90	OE1-N N-O	Phe114 Pro177 Glu236	Lys61 Ser28 Leu64
Conantokin Pr1	–	–	–	–	Glu236 Phe114 Pro177 Gln110	Ile15 His16 Asp3 Lys18
Conantokin Pr2	Gln110	Lys15	2.88	OE1-NZ	Glu236 Pro177 Phe114	Glu14 Gly1 Ala6
Conantokin Pr3	Gln110	Lys7	2.96	OE1-NZ	Ile111 Phe114 Pro177	Trp8 Glu2 Glu4
Conantokin T	Glu236	Arg13	2.69, 2.71	OE2-NE, OE2-NH2	Ile111	Glu16
Conantokin R	Gln110 Ile111	Lys73 Lys87	2.13 3.35	OE1-N O-NZ	Phe114 Pro177 Glu236	Glu95 Lys99 Asn62
Conantokin R1A	Gln110 Glu236	Leu7, Leu9, Thr5 Thr65	2.22 2.15, 2.80 1.79 3.14	OE1-N N-O, NE2-O NE2-O OE1-N	Ile111 Phe114 Pro177	Ile19 Arg63 Thr56
Conantokin R1B	–	–	–	–	Gln110 Ile111 Pro177 Glu236	Pro12 Ala77 Asp63 Thr76
Conantokin R1C	Gln110 Glu236	Val14 Ser52	2.47 2.54	OE1-N OE2-OG	Ile111 Phe114 Pro177	Gln65 His17 Leu49
Conantokin Br	Gln110	Ala94	1.93	NE2-O	Ile111 Pro177 Glu236	Asn59 Ile82 Asp77
Conantokin P	Glu236 Ile111	Leu42, Ala41 Gln89	1.27, 2.71 3.07	OE1-N, OE1-N N-O	Gln110 Phe114 Pro177	Thr30 Leu3 Ser21

**Fig. 6:**
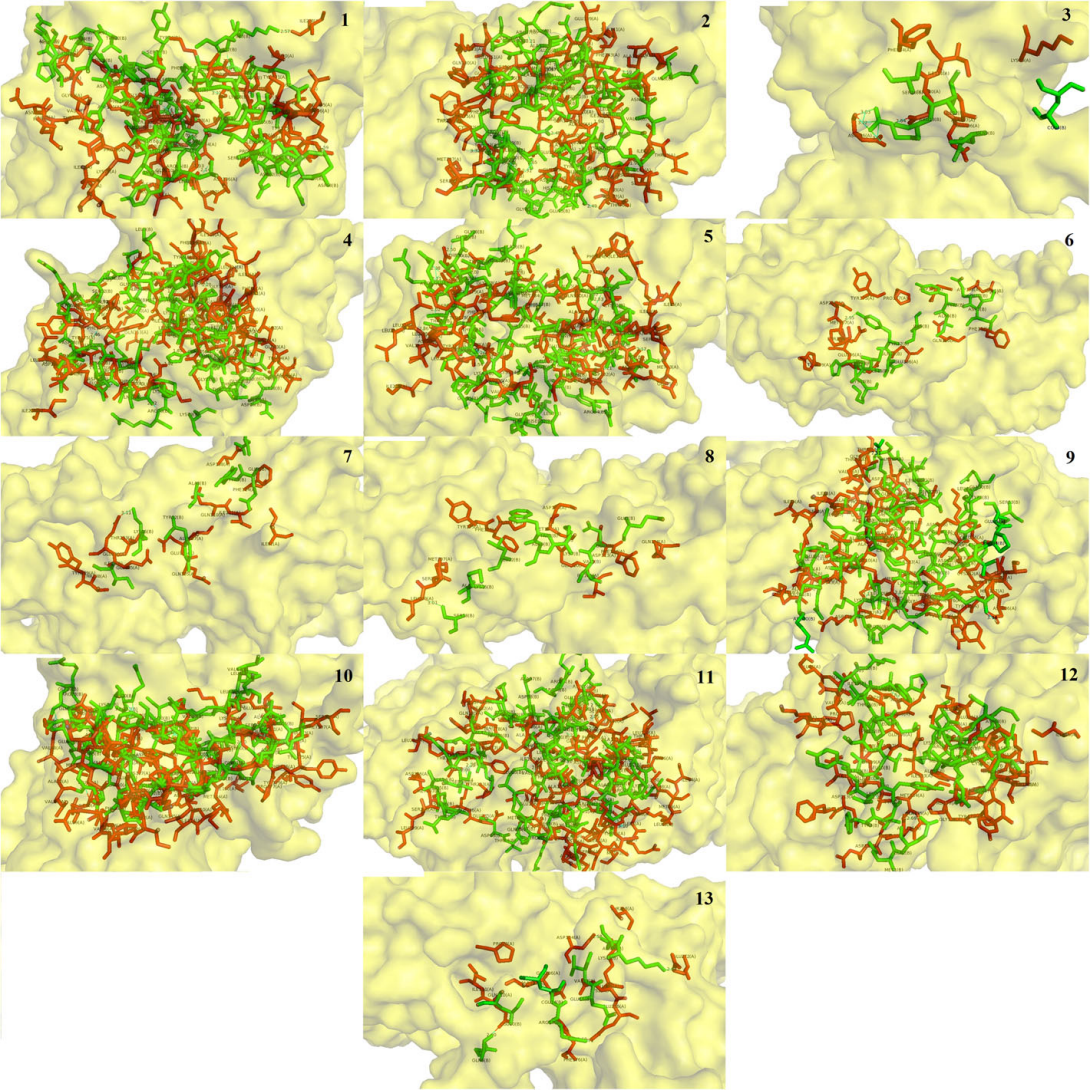
Three-dimensional binding representation of NMDAR (rats) with each conantokin ligand: (**1**) conantokin Br, (**2**) conantokin E, (**3**) conantokin G, (**4**) conantokin L, (**5**) conantokin P, (**6**) conantokin Pr1, (**7**) conantokin Pr2, (**8**) conantokin Pr3, (**9**) conantokin R, (**10**) conantokin R1B, (**11**) conantokin R1C, (**12**) conantokin R1A, (**13**) conantokin T. Receptor chain (red), ligand chain (green), ligand-receptor complex surface (yellow)

It has been observed that likewise for human NR2B, Glu236 and Gln110 residues of NR2B subunit in rats were found interacting with the residues of every conantokin ligand. Ile111, Phe114 and Pro177 were mostly found in hydrophobic interactions. This binding pattern was conserved among all the conantokins showing their similarity in potency for the NR2B subunit. The binding pattern of all conantokins for NMDAR in rats is shown in Fig. 6.

**Fig. 7:**
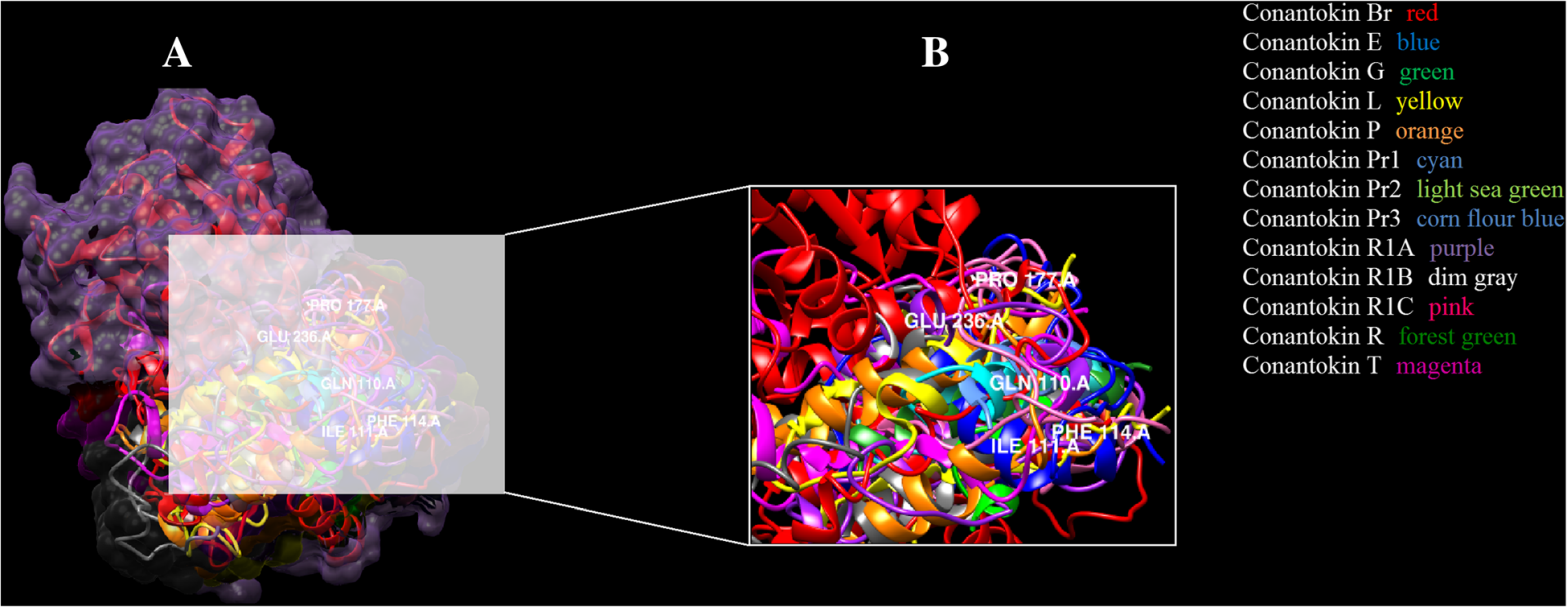
**a** Conantokins in complex with the NMDAR (rats). **b** Active residues in the binding pocket highlighted (white) of NMDAR with conantokin ligands. Three-dimensional binding representation of all toxins from the ligand dataset: conantokin Br (red), conantokin E (blue), conantokin G (green), conantokin L (yellow), conantokin P (orange), conantokin Pr1 (cyan), conantokin Pr2 (light sea green), conantokin Pr3 (cornflour blue), conantokin R1A (purple), conantokin R1B (dim gray), conantokin R1C (pink), conantokin R (forest green), conantokin T (magenta) with the NMDA receptor in rats

The binding pocket of conantokins with the receptor is highlighted in Fig. 7. As in the case of NMDAR in rats, Fig. 7 clearly demonstrates that each conantokin occupies the same binding pocket as NMDAR in humans.

### Energy values

**Fig. 8:**
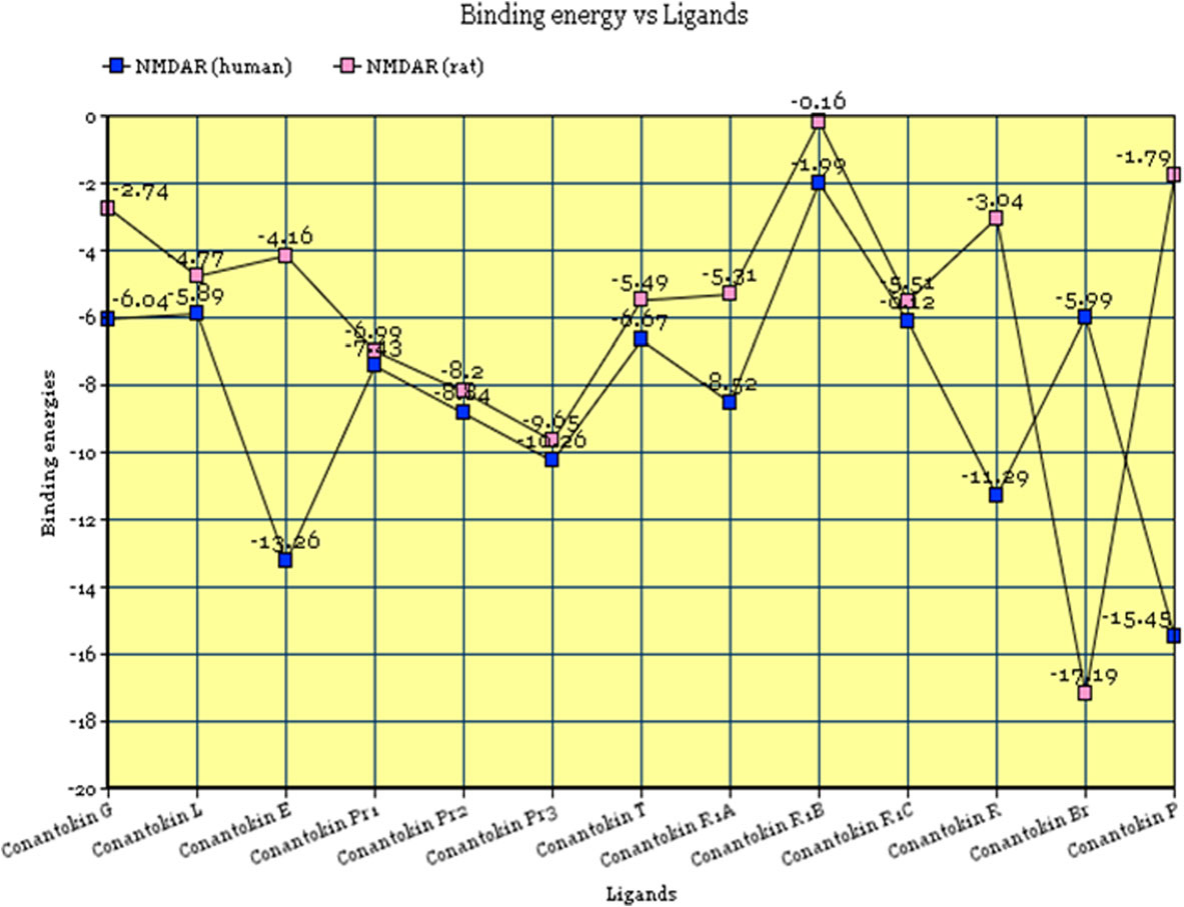
Graphical plot of binding energies versus toxin ligands

The overall stability of a complex is directly associated with the free energy of that complex. Lower energy values suggest the presence of a high binding affinity between the ligand and the receptor. It is of substantial importance to highlight each complex’s free energy in order to evaluate the successful complex formation. The successive energy values for the ligand-receptor complex formed by each toxin with the NMDA receptor in both humans and rats are shown in Fig. 8.

The energy values for all the complexes fall below the value of 0, as clearly observed from the graphical representation. This suggests the formation of relatively stable complexes between NMDA receptor in both rats and humans with all conantokins. NMDAR in humans showed most affinity for conantokin P, conantokin E, conantokin R and conantokin Pr3, whereas NMDAR in rats showed more affinity for Conantokin Br and Conantokin Pr3.

### Comparison of reported residues for NMDAR to docking results

A comparative analysis was performed of the docking results of NMDA receptors in both humans and rats with all the conantokins. The reported residues were inspected to verify if the docking results of this study were indeed in line with the reported information regarding the binding site residues of NR2B subunit of NMDAR. It is clearly displayed in Table 4 that the binding site residues of NMDAR reported in the literature were achieved in the docking experiments for both humans and rats.

**Table 4:** Comparative tabulation of binding residues of reported NMDAR versus post-docking results

Reported NMDAR binding site residues	Docking NMDAR binding residues (rat)	Docking NMDAR binding residues (human)
Gln110	Gln110	Gln110
Ile111	Ile111	Ile111
Phe114	Phe114	Phe114
Pro177	Pro177	Pro177
Glu236	Glu236	Glu236

## Conclusions

Conantokins had been reported as selective antagonists of NR2B subtype of the NMDA receptor. The NR2B subunit is directly involved in the excitotoxicity caused by the over-exposure to glutamate neurotransmitter. Therefore, inhibiting its activity has become a possible target for treatment of Alzheimer’s. We designed an in silico study and analysis of the binding interaction of conantokins with the NMDA receptor in both humans and rats. After the binding site residues for the receptors were understood, docking studies were performed and the residues achieved via docking were compared to the binding residues reported in the literature. The similarity of the results suggests the potential successful binding of the toxin ligands with both the receptors, and their subsequent function as their antagonists. Our results helped us to demonstrate the potential of these receptors in complex with the conantokins for the symptomatic treatment of Alzheimer’s patients.

## Abbreviations

CNS: Central nervous system
MSA: Multiple sequence alignment
NMDA: N-methyl-D-aspartate
NMDAR: NMDA receptor
NR2B: N-methyl D-aspartate receptor subtype 2B
RSCB: Research collaboratory for structural bioinformatics
RSMD: Root mean square deviation
SAVES: Structure analysis and verification server
